# Supplementary material for: Retinal Thickness Correlates with Cerebral Hemodynamic Changes in Patients with Carotid Artery Stenosis
Source: Brain Sci. 2022 Jul 25;12(8):979. doi: 10.3390/brainsci12080979 (PMC9331379; doi:10.3390/brainsci12080979)
Supplement: Supplementary file 1 [file brainsci-12-00979-s001.zip › brainsci-1805567-supplementary.pdf]

Supplementary Table S1. Demographics and ophthalmic information of all participants.

	CAS	Controls	*p*-Value
	37	37	
Age, years	63.95 ± 11.05		0.29
Gender, males	32	12	
Hypertension, n	22	17	0.25
Diabetes, n	9	9	1.0
Dyslipidemia, n	6	5	0.748
RNFL, μm	30.46 ± 3.28	33.36 ± 1.06	0.035
GCIPL, μm	68.58 ± 5.23	75.46 ± 3.27	<0.001
CVI	0.29 ± 0.06	0.30 ± 0.05	0.046
CVV	0.23 ± 0.09	0.29 ± 0.07	0.031
VA, logMAR $^{\Psi}$	0.17 ± 0.20	0.02 ± 0.06	<0.001

p-values for OCT/OCTA parameters were adjusted for age, gender and vascular risk factors (hypertension, diabetes and dyslipidemia). RNFL: retinal nerve fiber layer; GCIPL: ganglion cell-inner plexiform layer; CVI: choroidal vascular index; CVV: choroidal vascular volume; LogMAR: logarithm of minimum angle resolution; $^{\Psi}$ ANOVA.

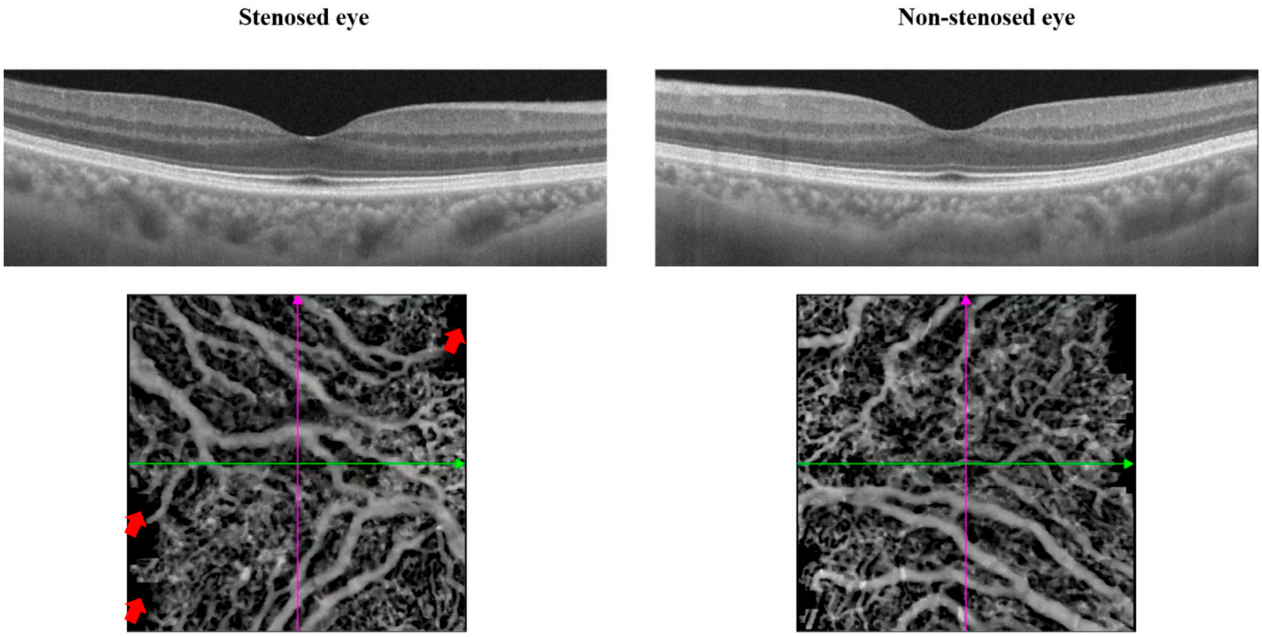

Supplementary Figure S1. Illustrative images of OCT/OCTA images of a CAS patient. Loss of choroidal vessels (red arrow) could be seen in stenosed eye compared to the non-stenosed eye.
